# The Relation of Scientific Creativity and Evaluation of Scientific Impact to Scientific Reasoning and General Intelligence

**DOI:** 10.3390/jintelligence8020017

**Published:** 2020-04-15

**Authors:** Robert J. Sternberg, Rebel J. E. Todhunter, Aaron Litvak, Karin Sternberg

**Affiliations:** Department of Human Development, College of Human Ecology, Cornell University, Ithaca, NY 14853, USA; rjt93@cornell.edu (R.J.E.T.); abl89@cornell.edu (A.L.); karin.sternberg@gmail.com (K.S.)

**Keywords:** intelligence, scientific impact, scientific reasoning, scientific creativity, fluid intelligence

## Abstract

In many nations, grades and standardized test scores are used to select students for programs of scientific study. We suggest that the skills that these assessments measure are related to success in science, but only peripherally in comparison with two other skills, scientific creativity and recognition of scientific impact. In three studies, we investigated the roles of scientific creativity and recognition of scientific impact on scientific thinking. The three studies described here together involved 219 students at a selective university in the Northeast U.S. Participants received assessments of scientific creativity and recognition of scientific impact as well as a variety of previously used assessments measuring scientific reasoning (generating alternative hypotheses, generating experiments, drawing conclusions) and the fluid aspect of general intelligence (letter sets, number series). They also provided scores from either or both of two college-admissions tests—the SAT and the ACT—as well as demographic information. Our goal was to determine whether the new tests of scientific impact and scientific creativity correlated and factored with the tests of scientific reasoning, fluid intelligence, both, or neither. We found that our new measures tapped into aspects of scientific reasoning as we previously have studied it, although the factorial composition of the test on recognition of scientific impact is less clear than that of the test of scientific creativity. We also found that participants rated high-impact studies as more scientifically rigorous and practically useful than low-impact studies, but also generally as less creative, probably because their titles/abstracts were seemingly less novel for our participants. Replicated findings across studies included the correlation of Letter Sets with Number Series (both measures of fluid intelligence) and the correlation of Scientific Creativity with Scientific Reasoning.

## 1. Introduction

Science-technology-engineering-mathematics (STEM) reasoning is an important aspect of daily life, not just for scientist and engineers, but for everyone. People are constantly being besieged not only by bogus scientific claims that some believe are true—for example, supposedly scientific studies claiming healthfulness of various kinds of foods (often funded by food-production companies)--but also by real scientific claims (such as supporting the importance of timely administration of childhood vaccines). True scientific claims are disputed by various celebrities, including actors, religious leaders, politicians, pop vocalists, and many others. For the past several years, we have stressed the importance of studying what we believe are the misunderstood processes of STEM reasoning (Sternberg and Sternberg 2017; Sternberg et al. (2017, 2019)) that are needed for making accurate scientific claims to distinguish them from the many bogus claims one finds in the media and elsewhere (Kaufman and Kaufman 2019; Shermer 2002). 

The consequences of failing to understand STEM principles and how people reason about them are great. Indeed, the consequences reach right up to the top: As this article is being written, even the presidents of some nations are climate-change deniers (Worland 2019). Denial of human-caused climate change has consequences throughout the world, such as rising temperatures, increased storm activity, and increasingly rapid depletion of potable water in some areas (The Guardian 2018; Kolbert 2019; Rettner 2019; Xia 2019). 

In our past research, we have learned a number of facts about STEM reasoning, some of which are contrary to popular conceptions (see Sternberg and Sternberg 2017; Sternberg et al. (2017, 2019)). Here is what we have found: First, reasoning about STEM seems to have a very weak and inconsistent relationship with general intelligence. Second, in contrast, different aspects of STEM reasoning have a moderate to strong relationship with each other. In particular, tests of hypothesis generation, experiment generation, drawing conclusions, evaluating teaching, and reviewing articles all appear to measure a core set of skills of scientific reasoning that cluster together factorially. Third, as might be expected, tests of inductive reasoning, such as letter sets, number series, and the SAT/ACT also measure a core set of skills that cluster factorially. Fourth, the factors for the two sets of clusters (scientific reasoning versus general intelligence as measured by conventional psychometric tests) are typically distinct (see details in Sternberg and Sternberg 2017; Sternberg et al. (2017, 2019)). Fifth, although the early studies examined only reasoning in psychological science (see Sternberg and Sternberg 2017; Sternberg et al. 2017), a later study (see Sternberg et al. 2019) showed that the results conceptually replicate and extend to reasoning in other scientific fields as well. Sixth, assessments of skills in evaluating quality of teaching correlate and factor with the scientific-reasoning measures rather than with the general-intelligence-based measures (see Sternberg et al. 2017). Seventh, when the scientific-reasoning measures are presented in multiple-choice format rather than in free-response format, their correlation with conventional tests measuring general intelligence increases (see Sternberg et al. 2019). Eighth, by implication, failing to measure skills in actual STEM reasoning may distort various kinds of admissions processes for STEM educational programs (graduate or even undergraduate). Admissions offices tend to focus on identifying students who are high in general intelligence and good at taking multiple-choice tests, but not necessarily adept in the STEM reasoning processes that the students will need to be fully successful in STEM educational programs.

These findings are important for the simple reason that proxy tests for general intelligence—or measures of so-called general mental ability (Sackett et al. 2020)—are, in fact, used regularly for admission to many undergraduate and graduate programs in STEM fields (Posselt 2018). Yet, the results suggest that the cognitive skills tapped by such tests are not at the core of scientific reasoning and even, in some cases, may be peripheral to it.

In our previous research, we tried to measure what we believed to be core scientific-reasoning skills, namely, generating alternative hypotheses, generating experiments, and drawing conclusions, as well as core skills for reasoning about quality of teaching. We also assessed skills involved in reviewing and editing scientific articles. However, less than adequately measured in our chosen set of skills were two sets of related skills that are particularly important in scientific research: those involved in scientific creativity and those involved in recognition of scientific impact. In our current studies, we sought to measure higher levels of scientific thinking skills than we did in our previous studies.

The first set of skills, those involved in scientific creativity, are essential in scientific thinking (Kaufman and Sternberg 2019; Simonton 2003; Sternberg 2019; Sternberg and Kaufman 2018) and in differentiating typical scientific thinkers from great ones (Simonton 2004). These skills include generating hypotheses, generating experiments, and drawing conclusions. To conduct research, one must be able to successfully complete each of these steps: generate initial hypotheses while considering alternative hypotheses (to ensure that there are indeed alternatives); generate experiments (to ensure that the research can yield strong conclusions); and draw conclusions (to make sure the researcher understands what the data are telling him or her). For example, in our subtest of Generating Experiments, we had participants generate experiments, but we, as researchers, provided them with each scientific problem and a corresponding hypothesis. The participants’ task was to design an experiment in order to test the presented hypothesis regarding the scientific problem. In many scientific-reasoning situations, at least among academic STEM professionals, the scientist her- or himself is the one who generates the problem and the hypothesis regarding the solution to the problem. Therefore, we believe it is important to extend our previous work to include a higher level of creativity, where the problem and hypothesis are participant-generated rather than researcher-generated. To this end, we created a new assessment called *Scientific Creativity*.

The second set of skills involves differentiating meaningful, high-impact scientific research from research of lower impact (Sternberg 2018d; Sternberg and Gordeeva 1996). These skills are important because one could be a good experimentalist, but for trivial research. For example, someone could design an experiment comparing recall for five-letter words to recall for 6-letter words, but the research likely would be trivial and have almost no impact. Some years back, Tulving and Madigan suggested that much scientific research is, in fact, scarcely worth doing and has little or no impact (Tulving and Madigan 1970). Impact provides a major heuristic benefit of scientific research, serving to generate further research on a given topic and sometimes opening up new, related topics for exploration (Sternberg 2016; Sternberg 2018b). It is often difficult to predict the impact of scientific research *before* the research is done, but measures exist to assess scientific impact *after* the research is done. To this end, we created a new assessment called *Scientific Impact*. We called upon two such measures for assessing scientific impact. 

Our first measurement of high impact involved inclusion of scientific work in prominent textbooks. Authors of scientific textbooks have to be very selective in the work they include in their texts because of the limited space they have to describe contributions to the field. Hence, the authors need to consider which studies they, as experts in their field, consider to be those of highest impact. They rely on their own judgment, of course, but also on the judgments of editors in their field and of other textbook authors. In order to measure students’ ability to differentiate between high- and low-impact research, we first provided students with a title and an abstract of each scientific work. Participants had to indicate whether they thought the work was high-impact (i.e., cited consistently in multiple major textbooks) or low-impact (not cited in major textbooks). Others also have used citations as measure of scientific impact (e.g., Dennis 1958; Zuckerman 1977).

The second measurement of high impact involved scientific work that has been highly cited in the scientific literature, without regard to whether it has been highly cited in textbooks (see also Feist (1997) and Liu et al. (2018)). As a basis for determining citations, we used the database SCOPUS, a science-citation index that is widely used in scientific research. SCOPUS describes itself as drawing on 1.4 billion cited references dating back to 1970. It has additional entries going back even further than that. It is probably the most well-known and respected data base for evaluating impact of work and was recently used in a major article to evaluate who are the 100,000 most widely cited scientists in the world (Ioaniddis et al. 2019).

Scientific impact is key to scientific contribution (Sternberg 2003a, 2003b; Sternberg 2018b). It helps to distinguish great scientists from not so great ones. Scientists who perform high-impact scientific research not only design scientifically-sound empirical studies, but also studies that are heuristically valuable to the field and hence are cited again and again (Sternberg 1997). 

This article extends our previous work in looking beyond our past scientific-reasoning measures to important aspects of scientific creativity and recognition of scientific impact. With regard to the latter, we recognize an important difference between recognition of impact and designing research that later will have impact. However, it is almost impossible to predict in advance which ideas will have great impact, and moreover, it is unrealistic to expect college-student participants to be in a position to design studies that later will be high-impact in science. Thus, we used a measure of *recognition* of scientific impact of ideas rather than of *generation* of scientifically impactful ideas. The latter measure would not be realistic in the context of our work with undergraduate students as participants.

## 2. Theoretical Basis

The theoretical basis for this work is the theory of successful intelligence (Sternberg 2018c, 2020; Sternberg et al. 2016). In this theory, creative intelligence combines with analytical intelligence and practical intelligence to provide a basis for understanding and predicting human thinking and behavior. In particular, there are seven metacomponents (executive processes) involved in scientific (and other forms of) thinking: (1) recognizing the existence of a problem; (2) defining the problem; (3) mentally representing the problem; (4) allocating resources to problem solution; (5) formulating a strategy for solving the problem; (6) monitoring problem solving; and (7) evaluating the problem solving. The previous studies we have done (see Sternberg and Sternberg 2017; Sternberg et al. (2017, 2019)) have emphasized reasoning and problem solving as realized in the latter metacomponents (the metacomponents numbered 3–7 above). But prior to problem solving, in science as in everyday life, there is a phase that is sometimes labeled problem-finding. This phase includes the first two metacomponents described above (i.e., the metacomponents numbered 1–2 above), namely, problem recognition and problem definition. These metacomponents involve realizing that there is a problem to be solved and figuring out what the problem is (usually prior to actually solving it, but sometimes as a result of redefining a problem that initially was incorrectly defined).

Conventional standardized tests emphasize problem solving (the latter five metacomponents) but place little, if any, emphasis on problem finding (the first two metacomponents). Yet, scientific thinking depends very heavily on the problem-finding, creative phase of research. Moreover, diverse recent models of problem-solving place substantial emphasis on problem finding (Abdulla and Cramond 2018; Abdulla et al. 2018; Arlin 1975; Arlin 1975–1976; Mumford and McIntosh 2017; Mumford et al. (1991, 2012); Simonton 2004). Our goal, therefore, was to place greater emphasis on this problem-finding phase in the current research.

In our past research (see Sternberg and Sternberg 2017; Sternberg et al. (2017, 2019)), we found that tests of scientific reasoning clustered with each other factorially, as did tests of fluid intelligence. However, the two groups of tests clustered relatively independently. We sought in this study to determine whether our new tests of scientific creativity and of prediction of scientific impact would cluster with the scientific-reasoning tests, the tests of fluid intelligence, with both, or with neither. We predicted that the test of scientific creativity would cluster with the scientific-reasoning tests. We expected the same for the test of prediction of scientific impact. We tested these predictions with both principal-components and principal-axis factor analysis (with results of the principal-components analysis shown) based on tables of correlations (with results shown). We first used analysis of variance to test for sex differences, simply to determine whether the data showed different means for men and women. This was done to ensure that any results we obtained were not due to mean differences between men and women, with sex operating as a moderator variable.

## 3. Study 1

### 3.1. Method

#### 3.1.1. Participants

A total of 59 participants were involved in the study, 23 males and 36 females. The average age was 20.2 and the range of ages was 18–26. All participants were students at a selective university in the northeast of the United States. The participants were students in behavioral-science classes that offered credit for experimental participation. 

#### 3.1.2. Materials

The materials were as follows:

Informed-consent form

1. Psychometric tests

We used two psychometric tests, which we also used in our previous research (see Sternberg and Sternberg 2017; Sternberg et al. (2017, 2019)) and which we found to be both reliable and construct valid.

(a) *Letter Sets.* For each item, participants saw five sets of four letters. They had to circle one set of letters that did not belong with the other four sets. For example, it might be that four sets of letters each had one vowel and one set of letters had no vowels. This test, with 15 items, was timed for 7 min. The test measures the fluid aspect of general intelligence.
(b) *Number Series.* Participants saw series of numbers. They had to indicate which number came next in each series. For example, they might be given a series of numbers in which each successive number was a multiple of 3 of the previous number. They then would have to figure out that the rule was “multiples of 3” and indicate the next multiple of 3. They wrote down what they believed to be the correct number. This test, with 18 items, was timed for 7 min. The test, like Letter Sets, measures the fluid aspect of general intelligence.

2. Assessing Scientific Impact

In this assessment, students were asked to evaluate scientific impact. “High-Impact” titles and abstracts were from articles that were cited in at least two of three major introductory-psychology textbooks: Myers (2011). *Myers’ Psychology* (2nd ed.). New York, NY: Worth; Weiten (2011). *Psychology: Themes and Variations* (9th ed.). Belmont: Wadsworth Cengage Learning; and Coon and Mitterer (2013). *Introduction to Psychology: Gateways to Mind and Behavior* (14th ed.). Boston: Cengage Learning. “Low-Impact” titles and abstracts were from articles that had been cited zero times in the textbooks and also less than five times in SCOPUS (average = 1.3 citations). 

There were ten high-impact and ten low-impact items. So that students would have some kind of reference point, we told them that half the items were high-impact, and half, low-impact. This instruction was to avoid a situation where, having no reference point, they viewed all or almost all the title/abstract combinations as either high or low in impact.

The titles and abstracts from both versions covered the following topics, with equal numbers of abstracts from each topic: medicine, neuroscience, biology, and behavioral sciences, with all research topics relevant to psychology. The participants were asked five questions about each title and its corresponding abstract. 

Here are the instructions and an example of an item we actually used:

“In psychological science, some studies have high impact and are cited many times. Other studies have low impact and are hardly cited at all. We are seeking to determine whether students, after reading abstracts of studies, can determine whether particular studies are high-impact or low-impact. For each of the following studies, we would like to ask you five questions. 

(1) Do you believe this study to be high-impact—cited many times—or low-impact—cited very few times? 

If you believe the study to be **high impact**, write an “**H**.” 

If you have the study to be **low impact**, write an “**L**.”

There are 10 high impact abstracts and 10 low impact. At the end, you may want to count how many times you put “H” and “L”. It should be 10 times for each.

(2) How confident are you in your rating?

If you have **high confidence** in your rating, write a “**3.**” 

If you have **medium confidence** in your rating, write a “**2.**” 

If you have **low confidence** in your rating, write a “**1.**”

For the three following questions, please rate your answer on a scale of 1 to 3, as you did for the previous question. For example, for “How creative do you believe this work to be?”, if you believe the work to be **highly creative**, write a “**3**”, if you believe this to be **somewhat creative** work, write a “**2**,” and if you believe this to be only **slightly creative** work, write a “**1.**”

(3) How creative do you believe this work to be?

3 = highly creative, 2 = somewhat creative, 1 = slightly creative

(4) How scientifically rigorous do you believe this work to be?

3 = highly rigorous, 2 = somewhat rigorous, 1 = slightly rigorous

(5) How practically useful do you believe this work to be in day-to-day life?

3 = highly practically useful, 2 = somewhat practically useful, 1 = slightly practically useful

On the next several pages, you will find various abstracts from papers that have been highly cited or have been rarely cited. They are in no particular order. Please answer the questions accordingly.”

1. ‘Can You See the Real Me? Activation and Expression of the “True Self” on the Internet’.

‘Those who feel better able to express their “true selves” in the Internet rather than face-to-face interaction settings are more likely to form close relationships with people met on the Internet. Building these correlational findings from survey data, we conducted three laboratory experiments to directly test the hypothesized causal role of differential self-expression in Internet relationship formation. Experiments 1 and 2, using a reaction time task, found that for the university undergraduates, the true self-concept is more accessible in memory during Internet interactions, and the actual self more accessible during face-to-face interactions. Experiment 3 confirmed that people randomly assigned to interact over the Internet (vs. face-to-face) were better able to express their true-self qualities to their partners.’”

[Quoted from Bargh et al. (2002). Can you see the real me? Activation and expression of the “true self” on the Internet. *Journal of Social Issues, 58,* p. 33.] (Participants were not told the original source of the title and abstract.)

Participants then answered the questions as described above.

3. Scientific-Creativity 

The third kind of assessment we used was of scientific creativity. After evaluating the 20 titles and abstracts and answering the five questions about each published study, the participants answered three questions about a potential study they could design:

“What is a study about human behavior that you might like to design and conduct? What is a question about human behavior that you consider important that you would like to answer? How might you answer it through research?”

Because we never have used this assessment before, we describe the scoring guidelines for the creativity test here. Ratings were holistic with regard to scientific creativity:

0 = missing
1 = answer unsatisfactory
2 = minimally satisfactory; answers question but is weak
3 = highly satisfactory; goes a step beyond minimum
4 = good; well beyond satisfactory answer
5 = outstanding

The scores were partly based on whether participants addressed all of the parts of the study design question. We used a single rater because our previous work (see Sternberg and Sternberg 2017) had shown that a single rater yielded reliable results, with our reliabilities at 0.75 in the previous work for multiple raters, suggesting a reliability of at more than 0.60 and less than 0.75 for a single rater. The baseline score was a 3 (if a participant made an effort to answer each part and if their proposed study made sense/was plausible). If they went above and beyond with creative detail, they were given a 4. A 5 was creatively outstanding (and also rare).

4. Scientific Reasoning

The fourth kind of assessment, scientific-reasoning items, were taken from our previous research (see Sternberg and Sternberg 2017; Sternberg et al. (2017, 2019)). The scientific-reasoning assessments included items that tasked students with evaluating research as well as using their own research skills. They had to generate hypotheses, generate experiments, and finally, draw conclusions. Here are sample items from among those we used:

● Generating Hypotheses

Participants were given brief descriptions of situations and had to create alternative hypotheses to explain the behavior described in the vignettes. One example of a vignette, as used in our previous research, is:

“Marie is interested in child development. One day, she notices that whenever Laura’s nanny comes in to pick up Laura from nursery school, Laura starts to cry. Marie reflects upon how sad it is that Laura has a poor relationship with her nanny.

*What are some alternative hypotheses regarding why Laura starts to cry when she is picked up from nursery school by the nanny?”*

Quality of each answer was scored on a 1 (low) to 5 (high) scale.

● Generating Experiments

A second set of vignettes was also presented to the participants. The participants were given a description of a situation with hypotheses, and students were tasked with designing an experiment to test these hypotheses. Here is an example:

“Ella, a senior in college, observes that her roommate tends to perform better on an exam if she has had a cup of coffee beforehand. Ella hypothesizes that drinking coffee before taking an exam will significantly increase one’s exam performance. However, Ella does not know how to test this hypothesis.

Please suggest an experimental design to test this hypothesis and describe the experiment in some detail. Assume you have the resources you need to be able to do the experiment (e.g., access to students and their academic records, sufficient funds to pay subjects, etc.).”

Quality of each answer was scored on a 1 (low) to 5 (high) scale.

● Drawing Conclusions

A third set of vignettes was presented to participants with results of studies. Students were asked whether the conclusions drawn were valid (and if not, why not). Here is the first item presented:

“Bill was interested in how well a new program for improving mathematical performance worked. He gave 200 students a pretest on their mathematical knowledge and skills. He then administered the new program to them. After administering the program, he gave the same 200 students a posttest that was equal in difficulty and in all relevant ways comparable to the pretest. He found that students improved significantly in performance from pretest to posttest. He concluded that the program for improving mathematical performance was effective.

Is this conclusion correct? Why or why not?”

Quality of each answer was scored on a 1 (low) to 5 (high) scale.

● Demographic Questionnaire

A demographic questionnaire was administered at the end of the study asking about gender, age, ethnicity, grade-point-average (GPA) at their university, relevant SAT and ACT scores, and experience in research.

#### 3.1.3. Design

The design of the study was totally within-subjects. All participants completed all assessments. For analysis-of-variance purposes, the main independent variable was participant gender and the main dependent variables were scores on the measures as described above. For purposes of factor analysis, the goal was to determine the factors (latent independent variables) that could predict scores on the various assessments.

#### 3.1.4. Procedure

All studies were conducted in person at a large selective university in the Northeast. The materials were arranged in the following order: (1) consent form; (2) letter-sets test; (3) number-series test; (4) title/abstract evaluations; (5) scientific creativity—designing their own study; scientific reasoning: (6) generating hypotheses; (7) generating experiments; (8) drawing conclusions; (9) demographic questionnaire. Lastly, the experimenter passed out a debriefing sheet. The letter-sets test and the number-series test were timed. The research assistants timed the students, telling them when to begin and when to turn the page. The students had 7 min to complete as many of the letter-set problems as possible, as well as 7 min to complete as many of the number-series problems as possible. None of the other assessments had a time limit. The students were given either course credit or $20 for their participation.

### 3.2. Results

#### 3.2.1. Basic Statistics

Table 1 shows basic statistics. In the Scientific-Impact ratings, participants rated each study as either high or low in impact. They were scored as to whether they gave the correct answer or the incorrect answer. Hence, a chance score would be 50%. The first question we asked is whether the mean scientific-impact scores differed significantly differently from 50% for each of the high-impact and low-impact items. If the mean score did not differ significantly from 50%, then we would conclude that the task was too hard and that participants were answering items at random. As this is a new assessment, never used by us (and, to our knowledge, anyone else) before, it is important to establish that participants even were able to do the task. The results of our analyses were *z* = 13.60, *p* < 0.001 for the high-impact items and *z* = 11.71, *p* < 0.001, *for the* low-impact items. Thus, the test items apparently were meaningful to the participants and they could answer correctly at above-chance levels.

We performed simple *t*-tests to determine whether there were significant sex differences on the two main new measures of the study, Scientific Creativity and Scientific Impact. For Scientific Creativity, we found *t* (56) = −0.72, *p* = 0.478. For the Scientific Impact measure, we found *t* (56) = −0.01, *p* = 0.992. Thus, there were no significant differences between the sexes. This is consistent with previous results in our work, where we have failed to find sex differences for our measures (see Sternberg and Sternberg 2017; Sternberg et al. (2017, 2019)). (Note: Degrees of freedom are reduced because of participants who did not report their gender as male or female.)

We also examined whether there were differences in the ratings of Creativity, Scientific Rigor, and Practical Usefulness for the impact ratings. The significance test results were, for Creativity, *t* (58) = −5.93, *p* < 0.001, for Scientific Rigor, *t* (58) = 7.55, *p* < 0.001, and for Scientific Usefulness, *t* (57) = 13.00, *p* < 0.001. In other words, there was a significant difference in each case for the ratings of high- versus low-impact items. 

The surprise in these ratings is that the difference for Creativity ratings went in the direction opposite to that expected. Participants rated low-impact titles/abstracts as more creative. This pattern of ratings may have resulted because our participants, university students and mostly freshmen and sophomores, weighed novelty, a first factor in creativity, very heavily, and usefulness, a second factor in creativity, not so much. Some of the low-impact studies were indeed quite novel, with titles, for example, such as “The positive effects of physical training on life quality of a 92-year-old female patient with exacerbation of chronic heart failure” or “Is painting by elephants in zoos as enriching as we are led to believe?” but the studies nevertheless were perhaps not as practically useful as the high-impact studies.

#### 3.2.2. Reliabilities

The new scales in this assessment are Scientific Creativity and Scientific Impact. The reliability of the Scientific Creativity measure could not be computed because it comprised only one item. The reliability of the Scientific Impact measure was 0.69 (computed by correlating scores for the two half-tests--low-impact items correlated with scores for high-impact items and corrected by the Spearman-Brown formula). Guttman lambda-5 reliability was 0.69. Letter Sets and Number Series were timed and thus equal halves needed to take into account that many people did not finish. The Guttman lambda-5 reliability of Letter Sets was 0.69 and of Number Series was 0.77. Some people also did not finish the Scientific-Reasoning items. The lambda-5 reliability of Scientific Reasoning was 0.85.

#### 3.2.3. Correlations

Table 2 shows correlations among the most important variables in Study 1. The expanded table of correlations is presented in the Appendix (Table A1). It should be kept in mind that any conclusions drawn from these correlations are limited by the power of the correlational tests. That said, the patterns of correlations for the psychometric tests and creative measure are akin to those in earlier studies from our research group with much larger samples (Sternberg and The Rainbow Project Collaborators 2006). As always, one cannot draw any clear conclusions from nonsignificant correlations. There are four key results, we believe:

(1) Our new Scientific-Creativity scores did not correlate significantly with scores on any of the conventional ability tests (SAT, ACT, Number Series) except Letter Sets (*r* = 0.32, *p* < 0.05) or with scores on our Scientific Impact measure. However, our Scientific Creativity scores did correlate significantly with our total Scientific Reasoning score (*r* = 0.49, *p* < 0.01).
(2) Our new Scientific Impact measure also did not correlate significantly with any of the conventional ability tests but did correlate significantly with our Scientific Reasoning scores (*r* = 0.27, *p* < 0.05). As always, one cannot draw any clear conclusions from nonsignificant correlations.
(3) Our Scientific Reasoning measure (total score) further did not correlate significantly with any of the conventional ability tests.
(4) Surprisingly, the SAT and ACT scores did not correlate significantly with the Letter Sets and Number Series scores (see Appendix A), although the samples were reduced because not everyone took either the SAT or ACT, some took one test of the other, and some took both. Letter Sets did correlate significantly with Number Series (*r* = 0.38, *p* < 0.01); SAT Reading and SAT Math correlated significantly with each other (*r* = 0.39, *p* < 0.05) and SAT Math correlated significantly with Number Series (*r* = 0.49, *p* < 0.01).

#### 3.2.4. Factor Analyses

We did two types of factor analyses, principal-components analysis and principal-factor analysis, each of which makes slightly different assumptions about the nature of the data. The difference is whether a 1 (principal-components analysis) or a communality (principal-factor analysis) is placed in the diagonal of the correlation matrix to be factor-analyzed. The results are shown in Table 3. The factor analyses did not include SAT or ACT scores because too few participants had taken the tests and did not include undergraduate GPA because many of the participants were freshmen and thus did not yet have a university GPA.

The results of principal-components (and principal-factor analyses, not shown at the request of the editor) suggested largely the same things:

(1) A first factor comprised Letter Sets, Number Series, and Scientific Impact (the last more weakly in the principal-factor analysis).
(2) A second factor comprised Scientific Creativity and Scientific Reasoning.

### 3.3. Discussion

To summarize these results, there were no sex differences in the key measures but significant differences in ratings of Creativity, Scientific Impact, and Practical Usefulness for high- versus low-impact items. We found that high-impact studies were judged as *less* creative, more scientifically rigorous, and more practically useful than low-impact studies. 

Our new Scientific Creativity measure correlated significantly with our old Scientific Reasoning measures, as used in past research (see Sternberg and Sternberg 2017; Sternberg et al. (2017, 2019)). The new Scientific Impact measure related as well to our Scientific Reasoning measures. The new measures did not show significant correlations with the psychometric tests except for one correlation significant at the 0.05 level with Letter Sets. Factorially, Scientific Creativity and Scientific Reasoning drew on the same set of latent skills. 

Scientific Impact factored with the fluid-intelligence tests. This may be in part because the test was largely an analytical one—that is, participants did not create high-impact studies but rather analyzed which of already existing studies were high- (or low-) impact. So, the Scientific Impact measure may have drawn more on fluid intelligence than we had expected it would.

A surprise in the ratings was that the difference for Creativity ratings went in the direction opposite to that expected. This may be because our participants, university students and mostly freshmen and sophomores, weighed novelty, a first factor in creativity, very heavily, and usefulness, a second factor in creativity not so much. As noted, some of the low-impact studies were indeed quite novel, for example, “Itch Relief by Mirror Scratching: A Psychophysical study,” but they were of limited practical usefulness.

## 4. Study 2

Study 2 provided a kind of conceptual replication of Study 1. As described earlier, instead of using textbook references, SCOPUS citations were used to decide which publications were high-impact. Although the two measures are similar in some ways, they are distinctly different in other ways.

First, studies can be highly cited in in the scientific literature and hence have high citation counts through SCOPUS for reasons that are not necessarily positive. As examples, work might be cited for failure to replicate, or because the work is in a fad area at a given time, because the work presents a useful new method of data analysis, or because the results are highly controversial either scientifically or socially. In contrast, work cited in introductory-psychology textbooks tends to have been replicated (if replication has been attempted), generally has risen above temporal fads, and tends to be substantive rather than methodological. It may be scientifically controversial but perhaps is less likely to be socially controversial because of a preference of textbook publishers not to lose sales by offending potential users (Sternberg 2003b; Sternberg and Hayes 2018).

Second, studies cited in textbooks often tend to be those thought to be of most interest to the largely undergraduate audience for the textbooks. So, studies of interest to the readership may be over-represented. Highly technical articles of any kind would be less likely to be cited in textbooks.

Third, SCOPUS does not capture all scholarly citations. It covers many but not all journals. Hence, it is comprehensive but not complete. Google Scholar, for example, covers more sources than does SCOPUS.

### 4.1. Method

#### 4.1.1. Participants

In this study, there were 54 participants, 18 males and 36 females. (Three participants were eliminated because they failed to complete any items at all on at least one task—that is, they simply did not engage the tasks.) The average age was 20.6 and the range of ages was 18–29. None of the participants had been involved in Study 1. The participants were students in behavioral-science classes that offered credit for experimental participation. 

#### 4.1.2. Materials

There were 20 titles and abstracts in all. The “High-Impact” titles and abstracts were from articles that, according to SCOPUS, were cited over 1500 times. The average “High-Impact” article in Version 2 was cited 7009 times. The “Low-Impact” articles, according to SCOPUS, were cited less than 10 times, for an average of 1 time per published study. 

As in Study 1, the abstracts from both versions covered the following topics, with equal numbers of abstracts from each topic: medicine, neuroscience, biology, and behavioral sciences. The participants were asked five questions about each abstract. These questions were the same as in Study 1.

#### 4.1.3. Design and Procedure

The design and procedure were the same as in Study 1. The only substantive difference between the two studies were the titles and abstracts presented to the participants, who, of course, were also different from those in Study 1.

### 4.2. Results

#### 4.2.1. Basic Statistics

Table 4 shows basic statistics. As in Study 1, we computed whether the mean impact ratings for high- and low-impact items differed significantly from a chance score of 50%. For the high-impact items, the result was *z* = 14.50, *p* < 0.001. For the low-impact items, the result was *z* = 12.44, *p* < 0.001. That is, in both cases, the items were meaningful to the participants in that they performed above a chance level.

As in Study 1, we tested for sex differences in the Scientific Creativity and Scientific Impact measures, the measures that are new to this work. The results were not significant, *t* (52) = 0.87, *p* = 0.387 for Scientific Creativity and *t* (50) = 0.48, *p* = 0.635, for Scientific Impact.

Also, as in Study 1, we tested for the significance of differences in ratings of Creativity, Scientific Impact, and Rigor for the high- versus low-impact items. All three, as in Study 1, were statistically significant, with, once again, the low-impact studies being rated as less creative but more scientifically rigorous and practically useful than the high-impact studies: for Creativity, *t*(53) = −7.392, *p* < 0.001, for Scientific Rigor, *t*(53) = 8.340, *p* < 0.001, and for Practical Usefulness, *t*(53) = 13.134, *p* < 0.001. As in Study 1, the creativity ratings were a surprise, suggesting that the novelty of the low-impact studies may have played a role in their perceived creativity.

#### 4.2.2. Reliabilities

The reliability of the Scientific Creativity measure could not be computed because it comprised only one item. The reliability of the Scientific Impact measure was 0.78 (computed by correlating scores for the two half-tests--low-impact items correlated with scores for high-impact items and corrected by the Spearman-Brown formula). Split-half reliability was 0.68 (again correcting by the Spearman-Brown formula). Letter Sets and Number Series, as in Study 1, were timed and thus equal halves needed to take into account that many people did not finish. Some people also did not finish the Scientific-Reasoning items. The Guttman lambda-5 reliability of Letter Sets was 0.63 and of Number Series was 0.82. The lambda-5 reliability of Scientific Reasoning was 0.74.

#### 4.2.3. Correlations

There were five key results in the correlations, shown in Table 5. A full table of correlations is shown in the Appendix (Table A2). It should be kept in mind that any of these conclusions is limited by the power of the correlational tests. As always, one cannot draw any clear conclusions from nonsignificant conclusions.

(1) Scientific Creativity correlated significantly with Scientific Reasoning—Generating Experiments (*r* = 0.33, *p* < 0.05), which makes sense because both assessments required participants to generate experimental designs, with the former requiring participants to generate their own scientific problem and the latter providing the problem and hypothesis.
(2) Unlike in Study 1, Scientific Reasoning correlated significantly with SAT Reading (*r* = 0.33, *p* < 0.05).
(3) SAT Reading and Math correlated highly with each other (*r* = 0.77, *p* < 0.01); ACT Reading and ACT Math also correlated highly with each other (*r* = 0.53, *p* < 0.05). (SAT and ACT were generally taken by different participants, so the correlations between them are based on small *N’s* and are not meaningful.)
(4) Letter Sets and Number Series correlated moderately with each other (*r* = 0.32, *p* < 0.05). Letter Sets also correlated moderately with Scientific Reasoning—Conclusions (*r* = 0.29, *p* < 0.05) but it did not correlate significantly with Scientific Reasoning overall.
(5) Scientific Impact correlated with ACT-Reading (0.51, *p* < 0.05) but did not correlate significantly with any of the other psychometric tests.

#### 4.2.4. Factor Analyses

Principal-components, shown in Table 6, were used as in Study 1. (Principal-factor analysis showed similar patterns of results but are not shown at the request of the editor.) There were three factors, which were similar for both components and factors. Again, SAT, ACT, and undergraduate GPA were not included for lack of sufficient numbers of cases:

(1) The first factor was for the fluid-intelligence tests.
(2) The second factor was for Scientific Creativity and Scientific Reasoning.
(3) The third factor was a specific factor for Scientific Impact.

### 4.3. Discussion

The results for means were as in Study 1, with no sex differences in the key measures and significant differences in ratings of Creativity, Scientific Impact, and Practical Usefulness for high- versus low-impact items. Again, with regard to our new measure of Scientific Impact, higher impact studies were rated as less creative, more scientifically rigorous, and more practically useful. Scientific Creativity correlated significantly with Scientific Reasoning—Generating Experiments, but the correlations did not reach significance with the other Scientific Reasoning measures. This time there were three factors, with the fluid-intelligence tests again factoring together, the Scientific Reasoning and Scientific Creativity tests factoring together, and Scientific Impact as its own factor. 

We sought in this study to investigate scientific reasoning more broadly than in our previous studies. In particular, we introduced two new measures to examine aspects of scientific thinking, a Scientific Creativity assessment and a Scientific Impact assessment. We found that the Scientific Creativity measure clustered with the Scientific-Reasoning measures, as predicted. The Scientific Impact measure appears largely to tap into skills not measured by existing tests. The former required participants to formulate and design a scientific experiment. The latter required participants to rate whether particular title/abstract combinations were of high or low impact. The overall pattern of data suggests that these measures are useful ways of assessing scientific reasoning beyond the measures we have used previously (hypothesis generation, experiment generation, drawing conclusions, reviewing articles, and editing articles—see Sternberg and Sternberg 2017; Sternberg et al. (2017, 2019). We do not know how simpler creative tasks would have fared, such as divergent tasks that require participants to generate unusual uses of a paper clip or to complete a drawing. But we and others have argued in the past that such measures tend to tap into a somewhat more trivial or, at least, limited aspect of creativity than that required in STEM research (Sternberg 2017; Sternberg 2018a, 2018c, 2018e). Creativity tests measure divergent thinking in a way that is divorced from scientific and even most everyday contexts (Sternberg 2018e). This has been shown to be true from both social-psychological (Amabile 1996) and cognitive perspectives (Ward and Kolomyts 2019). 

## 5. Study 3

Studies 1 and 2 lacked the power for us to draw firm conclusions. We therefore did a third study, Study 3, which had a substantially larger number of participants than did the preceding two studies. But we also introduced another substantial change. Instead of providing participants with both titles and abstracts of high- and low-impact studies, we provided just the titles. We therefore addressed the question of whether it was possible just from the titles of studies for participants to infer whether the studies were high- or low-impact.

Although our methodology largely replicated that of the previous studies, except for the change in the Scientific Impact items, our main concerns were to clarify results from the two previous studies. In particular, our main concerns in this study were four-fold: (a) to replicate whether our new test of Scientific Creativity does indeed factor with our tests of Scientific Reasoning and thus can be added to a battery of scientific-reasoning tests (convergent validity); (b) to determine whether this test of Scientific Creativity continued to show discrimination with respect to the standard psychometric tests (discriminant validity); (c) to assess whether our modified test of assessing Scientific Impact would be answered at better than chance levels; (d) to determine the factorial loadings of the new Scientific Impact measure, which were not so clear in the longer items (including titles and abstracts) of the previous studies. 

### 5.1. Method

#### 5.1.1. Participants

A total of 106 participants were involved in Study 3, 25 males and 81 females. The average age was 19 and the range of ages was 18–22. All participants in this study were students at a highly selective university in the northeastern United States. Ethnicities were recorded as follows: 33% European or European American; 33% Asian or Asian American; 10% African or African American; 9% Hispanic or Hispanic American; 8% Other; 7% No Response.

#### 5.1.2. Materials

*Letter Sets* and *Number Series* were as in the previous studies.

(a) Scientific Impact Items

The main goal of this section of the study was to have participants evaluate scientific impact. To accomplish this, participants were given 20 items, which consisted of titles (but unlike in the previous two studies, no abstracts) of scientific articles of studies that have been published in refereed journals. Ten of the items were considered low impact by the definition that the article had been cited fewer than 10 times by other academics. The other 10 items were considered high impact by the definition that the article had been cited more than 1000 times. Citation numbers were according to Web of Science and Research Gate.

The participants were told that there were 10 of each type of item (low-impact and high-impact) in order to provide a reference point for them and to prevent participants from putting the same answer for all the items. For each item, participants had to indicate whether the article title was high-impact or low-impact. All aspects of science were covered, ranging from psychology to academic medicine. 

Additionally, participants were also asked to rate, on a scale from 1 to 3, confidence in their choice, creativity of the title, scientific rigor of the title, and practical usefulness of the title. The premise behind these additional questions was to find additional correlations. For example, there was interest in whether impact was correlated with people’s own ideas of creativity or if impact was correlated with people’s own definitions of usefulness. 

The questions asked for each item were the same as in the previous studies.

Here is an example of an item we used:

(1) “An investigation of pesticide transport in soil and groundwater in the most vulnerable site of Bangladesh 

Do you believe this study to be high-impact—cited many times—or low-impact— cited very few times (H or L)?”

Other questions were as in the previous studies.

(b) Scientific Creativity (Open-Ended):

This measure was as in the previous studies.

The Scientific-Reasoning items were also as in the previous studies:

(c) Scientific Reasoning: Generating Hypotheses: 

(d) Scientific Reasoning: Generating Experiments 

(e) Scientific Reasoning: Drawing Conclusions

(f) Demographic Questionnaire:

At the end of the study a demographic questionnaire was administered which asked for the following information: gender, age, year of study, GPA, SAT scores, ACT scores, GRE scores, experience in lab courses, number of articles read per month, and ethnicity. Participants were given a debriefing form that outlined the purpose of the study and asked for their consent to use, analyze, and possibly publish their data anonymously. All 106 participants agreed and provided their signature. 

#### 5.1.3. Design

For the overall design, the independent variable of gender was evaluated for each of the assessments. For the internal validation, test scores were used as observable variables to discover the latent variables underlying them.

#### 5.1.4. Procedure

All studies and testing were conducted in person by a proctor at a selective university in the northeastern United States. The materials were administered in the following order: (1) informed consent form; (2) letter-sets test; (3) number-series test; (4) title-impact evaluations; (5) scientific creativity—designing their own study; (6) generating hypotheses; (7) generating experiments; (8) drawing conclusions; (9) demographic questionnaire; (10) debriefing form. 

The Letter Sets test and Number Series test were both individually timed by a research assistant. Participants received seven minutes for each test and were told by the proctor when to turn the page and begin the test as well as when to stop writing and end the test. No other sections had time constraints. Participants received credit in their courses as compensation for their time.

### 5.2. Results

Our goal in this study was to determine what results from the previous two studies replicated and which did not, so our emphasis is on this replicability of key findings from those previous studies. We have included substantially more participants in this study in order to ensure more stability for the data in assessing the replicability of findings and to determine whether merely presenting titles was sufficient for participants to distinguish high- from low-impact studies. 

#### 5.2.1. Basic Statistics

Table 7 shows basic statistics for the study. First, we wanted to determine whether there were significant sex differences. There were no significant differences on any of the cognitive tests. Once again, we failed to find meaningful differences between men and women.

Second, we wanted to show that, on the impact ratings, participants scored better than chance. The mean score for the participants was 14.62. A chance score would have been 10. The standard deviation, as shown, was 2.75. The result was *z* = 17.11, *z* < 0.001. Thus, participants, on average, performed at a level that was clearly above chance. They could distinguish high- versus low-impact items merely by titles without the abstracts as provided in Studies 1 and 2.

Third, we wanted to determine whether the various ratings of items differed from one another. Creativity ratings were higher for low-impact items than for high-impact items. The two means were 20.28 for high-impact items and 20.81 for low-impact items. The difference was in the predicted direction, given the first two studies, but did not reach statistical significance, *t*(104) = −1.37, *p* = 17. For Scientific Rigor, the high-impact studies were rated as more scientifically rigorous than the low-impact ones, *t*(103) = 7.06, *p* < 0.001. For usefulness, the difference was significant, with the high-impact studies rated as more useful than the low-impact ones, *t*(103) = 15.92, *p* < 0.001. These latter two results were as in the previous studies.

#### 5.2.2. Reliabilities

For simplicity, and at a reviewer’s request, we used Cronbach’s alpha for all tests. Cronbach’s alpha was 0.64 for Letter Sets, 0.69 for Number Series, 0.58 for Scientific Impact, 0.78 for ratings of confidence, 0.75 for ratings of scientific creativity, 0.76 for ratings of scientific rigor, 0.70 for ratings of usefulness, and 0.78 for the test of Scientific Reasoning, these internal-consistency reliabilities were roughly comparable to the earlier studies reported above. For Scientific Creativity, the correlation between raters was 0.82. There was no need for inter-rater reliability for the Scientific Impact test because it was scored correct-incorrect.

At a reviewer’s request, we used two raters to determine whether the two raters would show commonality in their ratings for those tests in which ratings were applicable. The correlations between raters were 0.98 for Scientific Hypotheses, 0.89 for Generating Experiments, and 0.87 for Drawing Conclusions, indicating that the raters were largely consonant with each other. These correlations were similar to those in previous research (see Sternberg and Sternberg 2017; Sternberg et al. 2017). 

#### 5.2.3. Correlations and Factor Analyses

Table 8 shows the correlation matrix of the main variables. A more extensive correlation matrix including SATs as used in the factor analysis is in the Appendix (Table A3). Although we used SAT in these analyses, we also did factor analyses with ACT scores and by combining SATs and ACTs using a conversion table. The results were comparable. 

Table 9 shows the results of principal-components analysis. The results are similar although not identical to those of the preceding studies. In the principal-component analysis, the first rotated component was for three psychometric tests: SAT Reading, SAT Math, and Number Series. The second rotated principal component was for our Scientific Reasoning and Scientific Creativity measures. The third principal component was for Scientific Impact and Letter Sets. Principal-factor analysis yielded similar results.

### 5.3. Discussion

As before, there were no meaningful sex differences. Rated Scientific Rigor and Usefulness were rated higher for high-impact items than for low-impact items. Rated Scientific Creativity was not significantly lower for the High-Impact items.

Again, our Scientific Reasoning measures tended to cluster with our Scientific Creativity measure, our psychometric tests tended to cluster together (although this time Letter Series behaved somewhat differently), and our Scientific Impact measure did not show a clear factorial pattern with respect to the other measures. Both Letter Sets and Number Series showed significant correlations with our Scientific Reasoning measure, showing that general-intellectual processes presumably play some role in scientific reasoning, a conclusion that seems plausible given that almost all cognitive processing involves at least some degree of general intelligence. In this study, Scientific Reasoning was correlated with the Letter Sets and Number Series measures. These latter measures were correlated with SAT scores, as would be expected.

These results, for the most part, are consistent with our previous findings. Our main interest in this new work was with regard to our new measures of Scientific Creativity and of Scientific Impact. The former, Scientific Creativity, seems to fit in with our Scientific Reasoning battery. The latter, Scientific Impact, probably will need further convergent-discrimination validity analysis to figure out exactly what it measures.

## 6. General Discussion

The main replicated findings included the correlation of Letter Sets with Number Series (both measures of fluid intelligence) and the correlation of Scientific Creativity with Scientific Reasoning. The results suggest that the measure of Scientific Creativity we used fit very well with our previous Scientific Reasoning measures, perhaps because it directly measures creative thinking, as did our previous measures. The picture with the Scientific Impact measure is less clear. In Study 1, it factored with the fluid-intelligence tests; in Study 2, it factored on its own. In Study 3, it factored with Letter Sets in one analysis (principal-components) and did not cluster with it in another (principal-factor). 

On the one hand, recognition of scientific impact is important in scientific research, as those who fail to recognize it may be as apt to pursue trivial research as to pursue scientifically meaningful research. On the other hand, our task required analysis of impact rather than creative production of impactful ideas, which may be why the Scientific Impact measure did not factor as clearly with our other measures of scientific thinking. That said, we of course cannot guarantee and, in fact, seriously doubt that the studies we chose to represent the high-and low-impact domains were representative of all scientific research in those domains. For example, we did not choose any titles/abstracts that would have been largely incomprehensible to our audience of undergraduates because of their heavy use of highly technical terms. 

Our studies were limited in other ways as well. The samples were relatively small and limited to students in one university, we did not draw from all domains of scientific endeavor, and we used only a single item in our new Scientific Creativity measure (because of the amount of time it took participants to find a scientific problem to solve and then to state how they would solve it). Future research will address some of these issues. Most notably, we need to follow up on the finding of a negative correlation between rated creativity and impact, and plan to do a study where we define creativity for our participants along the usual lines of the definition of creativity in terms of novelty and usefulness (Simonton 2010; Lubart 2001; Kaufman and Sternberg 2019).

We believe that the new Scientific Creativity and Scientific Impact measures are worthy of further investigation. These measures provide further understanding of important aspects of scientific thinking. Our results suggest that investigators, just by considering the titles of their projects/articles, could make some prediction as to whether the studies are more likely to be high- or low-impact. Although we suspect that no one can guess well what studies will be highly cited, the titles do provide a good diagnostic as to which studies will be low-impact. The low-impact titles were generally ones for studies of very narrow problems or whose generalizability was confined to narrow geographic locations. 

Our goal is, ultimately, to show that scientific thinking in STEM disciplines is so important, and so different from the kinds of thinking involved in standardized tests, that it would behoove STEM programs, especially at the graduate level, to seek to assess not only general mental ability and knowledge base in the sciences, but also the core skills involved in scientific thinking, as measured by tests such as Hypothesis Generation, Experiment Generation, Drawing Conclusions, Scientific Creativity, and Scientific Impact. 

## Figures and Tables

**Table 1:** Descriptive Statistics Study 1.

Assessment	N	Mean	SD
Scientific Creativity	58	2.60	0.79
Letter Sets	59	10.19	2.51
Number Series	59	10.49	3.01
Scientific Reasoning--Hypotheses	59	6.59	2.91
Scientific Reasoning--Experiments	59	6.34	2.19
Scientific Reasoning--Conclusions	59	6.97	1.60
Scientific Reasoning (Total)	59	19.90	5.32
Impact Ratings High	58	7.34	1.38
Impact Ratings Low	58	7.17	1.92
Average of confidence ratings for high impact items	59	2.21	0.32
Average of confidence ratings for low impact items	57	2.16	0.35
Average of creativity ratings for high impact items	59	1.94	0.29
Average of creativity ratings for low impact items	59	2.24	0.35
Average of rigor ratings for high impact items	59	2.09	0.33
Average of rigor ratings for low impact items	59	1.74	0.25
Average of usefulness ratings for high impact items	59	2.36	0.29
Average of usefulness ratings for low impact items	58	1.70	0.27
Age	59	20.24	1.78
GPA	59	3.39	0.47
SAT Reading	40	702.25	70.51
SAT Math	40	734.75	49.30
ACT Reading	28	32.07	4.15
ACT Math	28	33.25	3.09
What is the number of lab courses you have taken?	55	2.18	2.39
How many scientific articles do you read per month?	58	5.55	8.64

**Table 2:** Key Correlations Study 1.

Assessment	Sci. Creat.	LS	NS	Sci Reas.	Sci Imp.
Sci. Creat.	1.00	0.32 *	0.09	0.49 **	0.07
Letter Sets	0.32 *	1.00	0.38 **	0.05	0.21
Num. Ser.	0.09	0.38 *	1.00	0.22	0.23
Sci. Reas.	0.49 **	0.05	0.22	1.00	0.27 *
Sci. Imp.	0.07	0.21	0.23	0.27 *	1.00

* *p* < 0.05; ** *p* < 0.01; all tests two-tailed; Note: Correlations in the table are presented with pairwise deletion.

**Table 3:** Study 1: Rotated Principal Components.

Assessment		Component
	**I**	**II**
Letter Sets	0.77	0.16
Number Series	0.77	0.04
Scientific Reasoning	0.15	0.84
Scientific Creativity	0.09	0.88
Scientific Impact	0.64	0.10
Extraction Method: Principal-component analysis		
2 Components Extracted Rotation Method: Varimax with Kaiser Normalization		
Rotation converged in 3 iterations Percentage of Variance Account for: 62%.		

Factors included for Eigenvalues >1. These were also the most interpretable factors.

**Table 4:** Descriptive Statistics Study 2.

Assessment	N	Mean	Std. Deviation
Scientific Creativity	54	2.46	0.77
Letter Sets	54	9.72	2.29
Number Series	54	10.63	3.13
Hypotheses	54	6.85	2.80
Experiments	54	6.39	1.90
Conclusions	54	6.24	1.16
Scientific Reasoning	54	19.48	4.06
Impact_high	53	7.72	1.47
Impact_low	53	7.55	1.60
Average of confidence ratings for high impact items	54	2.18	0.37
Average of confidence ratings for low impact items	54	2.19	0.39
Average of creativity ratings for high impact items	54	1.88	0.37
Average of creativity ratings for low impact items	54	2.34	0.33
Average of rigor ratings for high impact items	54	2.20	0.29
Average of rigor ratings for low impact items	54	1.74	0.29
Average of usefulness ratings for high impact items	54	2.47	0.29
Average of usefulness ratings for low impact items	54	1.74	0.31
Age	54	20.59	2.00
GPA	54	3.39	0.50
SATReading	38	702.89	67.50
SATMath	40	718.50	84.02
ACTMath	20	32.60	5.01
ACTReading	20	32.50	3.89
GRE	4	300.50	50.72
What is the number of lab courses you have taken?	52	1.90	1.76
How many scientific articles do you read per month?	50	6.06	11.40

**Table 5:** Key Correlations: Study 2.

Assessement	Sci. Creat.	LS	NS	Sci Reas.	Sci Imp.
Sci. Creat.	1.00	0.22	0.03	0.22	0.00
Letter Sets	0.22	1.00	0.32 *	0.16	−0.10
Num. Ser.	0.03	0.32 *	1.00	0.04	0.11
Sci. Reas.	0.22	0.16	0.04	1.00	0.14
Sci. Imp.	0.00	−0.10	0.11	0.14	1.00

* *p* < 0.05; ** *p* < 0.01; all tests two-tailed. Note: Correlations in the table are presented with pairwise deletion.

**Table 6:** Study 2: Rotated Principal Component Matrix.

Assessment	I	II	III
Letter Sets	0.75	0.31	−0.26
Number Series	0.86	−0.13	0.22
Scientific Reasoning	0.03	0.72	0.33
Scientific Creativity	0.05	0.78	−0.16
Scientific Impact	0.02	0.05	0.92
Extraction Method: Principal-component analysis.			
3 components extracted. Rotation Method: Varimax with Kaiser normalization. 78Rotation converged at 5 iterations. Components accounted for 73% of variance in data. Factors included for Eigenvalues >1. These were also the most interpretable factors.			

**Table 7:** Basic Statistics for Study 3.

Assessment	N	Mean	Standard Deviation
s	106	10.12	2.51
Number Series	106	10.58	2.92
LS+NS	106	20.70	4.45
Hypotheses	105	7.40	3.35
Experiments	106	6.80	1.38
Conclusions	105	6.91	1.47
Sci. Reas. Tot.	104	21.10	4.80
Impact_Low	106	7.09	1.61
Impact_High	105	7.51	1.61
Impact Total	105	14.62	2.75
Confidence_High	106	23.16	3.12
Confidence_Low	106	22.70	3.44
Creativity_High	106	20.28	3.22
Creativity_Low	105	20.81	3.81
Rigor_High	103	21.86	3.32
Rigor_Low	106	19.57	3.13
Practicality_High	103	24.68	2.96
Practicality_Low	106	18.58	3.41
Age	106	19.41	1.128
GPA	77	3.56	0.36
SATReading	60	712.67	55.54
SATMath	63	745.87	63.90
SAT/ACTCombined	96	64.63	5.39
ACTReading	48	32.79	2.91
ACTMath	49	33.00	2.82
LabCourses	105	1.76	1.75
Articles	101	3.81	5.55

**Table 8:** Correlation Matrix for Factor- Analyzed Variables.

Assessment	LS	NS	Sci. Rea.	Sci. Creat.	Impact
Letter Sets	1	0.26 *	0.32 *	0.04	0.21
Number Ser.	0.26 *	1	0.26 *	0.07	0.10
Sci. Reason.	0.32 *	0.26 *	1	0.32 *	0.08
Sci. Creativ.	0.04	0.07	0.32 *	1	0.06
Impact	0.21	0.10	0.08	0.06	1

* *p* < 0.05, ** *p* < 0.01.

**Table 9:** Rotated Principal Component Matrix.

		Component	
	I	II	III
Letter Sets	0.20	0.23	0.71
Number Series	0.73	0.20	0.19
Sci. Reason.	0.08	0.79	0.26
Sci. Creativity	0.02	0.80	−0.11
SATReading	0.73	−0.05	0.27
SATMath	0.83	−0.00	−0.22
Impact	−0.02	−0.07	0.76

Extraction Method: Principal Component Analysis; Rotation Method: Varimax with Kaiser Normalization; Rotation converged in four iterations, accounting for 64% of the variance in the data.

## Appendix A

**Table A1:** Correlations Study 1.

Assessment	Sci. Creativ.	GPA	SATRea	SATM	ACTRea	ACTMat	Let Sets	Num Ser	Hyp	Exper	Concl	SciRe Tot	Imp Hi	Imp Lo	Tot Imp
Sci. Creat	1.00	−0.16	0.23	0.00	0.30	0.36	0.32 *	0.09	0.36 **	0.46 **	0.34 **	0.49 **	−0.01	0.11	0.07
GPA	−0.16	1.00	0.36 *	0.39 *	0.50 **	0.20	−0.01	0.27 *	0.06	0.15	−0.12	0.06	0.23	0.08	0.16
SATR	0.23	0.36 *	1.00	0.39 *	0.82 **	−0.30	−0.03	0.11	0.22	0.11	0.11	0.20	0.11	−0.09	−0.01
SATM	0.00	0.39 *	0.39 *	1.00	0.49	0.53	0.25	0.49 **	0.06	0.19	−0.02	0.10	0.13	0.06	0.10
ACTR	0.30	0.50 **	0.82 **	0.49	1.00	0.34	0.00	−0.03	0.30	0.40 *	−0.15	0.29	0.16	−0.02	0.07
ACTM	0.36	0.20	−0.30	0.53	0.34	1.00	0.17	0.35	0.18	0.35	0.27	0.32	0.07	0.09	0.10
Let Sets	0.32 *	−0.01	−0.03	0.25	0.00	0.17	1.00	0.38 **	−0.13	0.22	0.10	0.05	0.12	0.23	0.21
Numb Ser.	0.09	0.27 *	0.11	0.49 **	−0.03	0.35	0.38 **	1.00	0.08	0.40 **	0.02	0.22	0.17	0.23	0.23
Hyp	0.36 **	0.06	0.22	0.06	0.30	0.18	−0.13	0.08	1.00	0.50 **	0.42 **	0.88 **	0.23	0.12	0.19
Exps	0.46 **	0.15	0.11	0.19	0.40 *	0.35	0.22	0.40 *	0.50 **	1.00	0.30 *	0.78 **	0.18	0.15	0.18
Concs	0.34 **	−0.12	0.11	−0.02	−0.15	0.27	0.10	0.02	0.42 **	0.30 *	1.00	0.65 **	0.21	0.32 *	0.31 *
Sci Reason Total	0.49 **	0.06	0.20	0.10	0.29	0.32	0.05	0.22	0.88 **	0.78 **	0.65 **	1.00	0.26 *	0.22	0.27 *
Impact HI	−0.01	0.23	0.11	0.13	0.16	0.07	0.12	0.17	0.23	0.18	0.21	0.26 *	1.00	0.53 **	0.83 **
Impact Lo	0.11	0.08	−0.09	0.06	−0.02	0.09	0.23	0.23	0.12	0.15	0.32 *	0.22	0.53 **	1.00	0.92 **
Sci Impact Total	0.07	0.16	−0.01	0.10	0.07	0.10	0.21	0.23	0.19	0.18	0.31 *	0.27 *	0.83 **	0.92 **	1.00

* *p* < 0.05; ** *p* < 0.01; all tests two-tailed; Note: Correlations in the table are presented with pairwise deletion.

**Table A2:** Correlations: Study 2.

Assessment	Sci. Creat	GPA	SATR	SATM	ACTR	ACTM	LS	NS	Hyp	Exp	Concl	Sci Reas	Imp Hi	Imp Lo	Sci Imp Tot
Sci. Creat.	1.00	0.07	0.19	0.05	−0.20	−0.11	0.22	0.03	0.02	0.33 *	0.17	0.22	0.00	0.00	0.00
GPA	0.07	1.00	0.32 *	0.20	−0.06	0.51 *	0.14	0.36 **	0.01	0.15	−0.18	0.03	−0.02	0.15	0.08
SATR	0.19	0.32 *	1.00	0.77 **	0.75 *	0.94 **	0.22	0.37 *	0.37 *	0.15	0.09	0.33 *	0.08	0.15	0.14
SATM	0.05	0.20	0.77 **	1.00	0.21	0.35	0.12	0.54 **	0.23	−0.01	0.10	0.17	−0.05	−0.06	−0.06
ACTR	−0.20	−0.06	0.75 *	0.21	1.00	0.53 *	−0.38	−0.03	0.28	0.27	−0.26	0.23	0.46 *	0.53 *	0.51 *
ACTM	−0.11	0.51 *	0.94 **	0.35	0.53 *	1.00	−0.01	0.64 **	−0.04	0.09	−0.54	−0.21	0.34	0.42	0.40
Letter Sets	0.22	0.14	0.22	0.12	−0.38	−0.01	1.00	0.32 *	0.07	0.07	0.29 *	0.16	−0.04	−0.13	−0.10
Num. Series	0.03	0.36 **	0.37 *	0.54 **	−0.03	0.64 **	0.32 *	1.00	0.02	0.05	0.01	0.04	0.14	0.06	0.11
Hyp	0.02	0.01	0.37 *	0.23	0.28	−0.04	0.07	0.02	0.00	0.19	0.17	0.83 **	0.22	0.06	0.15
Exp	0.33 *	0.15	0.15	−0.01	0.27	0.09	0.07	0.05	0.19	1.00	0.13	0.64 **	0.00	0.23	0.14
Concl	0.17	−0.18	0.09	0.10	−0.26	−0.53 *	0.29 *	0.01	0.17	0.13	1.00	0.46 **	−0.09	−0.08	−0.10
Sc. Reas. Tot	0.22	0.03	0.33 *	0.17	0.23	−0.21	0.16	0.04	0.83 **	0.64 **	0.46 **	1.00	0.12	0.12	0.14
Impac Hi	0.00	−0.02	0.08	−0.05	0.46 *	0.34	−0.04	0.14	0.22	0.00	−0.09	0.12	1.00	0.64 **	0.90 **
ImpactLow	0.00	0.15	0.15	−0.06	0.53 *	0.42	−0.13	0.06	0.06	0.23	−0.08	0.12	0.64 **	1.00	0.91 **
Sci. Imp. Tot	0.00	0.08	0.14	−0.06	0.51 *	0.40	−0.10	0.11	0.15	0.14	−0.10	0.14	0.90 **	0.91 **	1.00

* *p* < 0.05; ** *p* < 0.01; all tests two-tailed; Note: Correlations in the table are presented with pairwise deletion.

**Table A3:** Correlation Matrix for Factor- Analyzed Variables.

Assessment	LS	NS	Sci Reas	Sci. Creat.	SATReading	SATMath	Impact
Letter Sets	1	0.26 *	0.32 *	0.04	0.27 *	0.02	0.21
Number Ser.	0.26 *	1	0.26 *	0.07	0.38 **	0.41 **	0.10
Sci. Reason.	0.32 *	0.26 *	1	0.32 *	0.06	0.04	0.08
Sci. Creativ.	0.04	0.07	0.32 *	1	0.06	0.04	0.06
SATReading	0.27 *	0.38 **	0.06	0.06	1	0.39 **	0.15
SATMath	0.02	0.41 **	0.04	0.04	0.39 **	1	-0.04
Impact	0.21	0.10	0.08	0.06	0.15	-0.04	1

* *p* < 0.05; ** *p* < 0.01.
